# Assessing the relationship between river water pollution and the LULC composition of a basin in the Isthmus of Tehuantepec in Oaxaca, Mexico

**DOI:** 10.1007/s10661-024-13147-3

**Published:** 2024-10-11

**Authors:** Felipe-Omar Tapia-Silva, José García-Hernández

**Affiliations:** 1 Lab. Applied Geomatics, Hydrobiology Department, Autonomous Metropolitan University, Ciudad de Mexico, Iztapalapa Mexico; 2 Postgraduate in Geography, Center of Research in Environmental Geography, National Autonomous University of Mexico, Morelia, Michoacán Mexico; 3 University of Chalcatongo, Oaxaca, Mexico

**Keywords:** Random forest classification, Canonical correspondence analysis, Catchment areas, Water pollution indicators, LULC change analysis

## Abstract

Water pollution originating from land use and land cover (LULC) can disrupt river ecosystems, posing a threat to public health, safety, and socioeconomic sustainability. Although the interactions between terrestrial and aquatic systems have been investigated for decades, the scale at which land use practices, whether in the entire basin or separately in parts, significantly impact water quality still needs to be determined. In this research, we used multitemporal data (field measurements, Sentinel 2 images, and elevation data) to investigate how the LULC composition in the catchment area (CA) of each water pollution measurement station located in the river course of the Los Perros Basin affects water pollution indicators (WPIs). We examined whether the CAs form a sequential runoff aggregation system for certain pollutants from the highest to the lowest part of the basin. Our research applied statistical (correlation, time series analysis, and canonical correspondence analysis) and geo-visual analyses to identify relationships at the CA level between satellite-based LULC composition and WPI concentrations. We observed that pollutants such as nitrogen, phosphorus, coliforms, and water temperature form a sequential runoff aggregation system from the highest to the lowest part of the basin. We concluded that the observed decrease in natural cover and increase in built-up and agricultural cover in the upper CAs of the study basin between the study period (2016 to 2020) are related to elevated WPI values for suspended solids and coliforms, which exceeded the allowed limits on all CAs and measured dates.

## Introduction

Water pollution originating from land use and land cover (LULC) can disrupt river ecosystems (Guo et al., 2010), posing a threat to public health, safety, and socioeconomic sustainability (Batsaikhan et al., 2018; Halder & Islam, 2015). Different types of landscapes and specific sources of contamination (Fu et al., 2005; Liu et al., 2012; Xiao et al., 2016; Zhao et al., 2015) can produce pollutants that can lead to eutrophication in aquatic ecosystems. This can result in hypoxia, anoxia, frequent fish deaths, increased turbidity, loss of submerged aquatic vegetation, and changes in the food web structure (Boesch et al., 2001). The excessive use of phosphorus fertilizers in agricultural areas can cause phosphorus accumulation in the soil (Bennett et al., 2001) and surface waters, especially during intense precipitation (Shigaki et al., 2007). Excess nitrogen and phosphorus in water can stimulate abnormal algae growth, which prevents sunlight from reaching submerged aquatic vegetation that serves as food and habitat for various species.

Since the 1970s, remote sensing, GIS, and statistical techniques have been used to assess the interactions between terrestrial and aquatic systems (Xu et al., 2021). Numerous studies have investigated the relationship between landscape composition and surface water quality (Mashala et al., 2023; Gani et al., 2023; Luo et al., 2020; González-Rivas et al., 2020; Li et al., 2019; Wang & Zhang, 2018; Xiao et al., 2016; Yu et al., 2016; Johnson & Gage, 1997). The following analytical methods help to understand this relationship: regression and correlation analysis (Gani et al., 2023; Li et al., 2019; Liu et al., 2012; Wang & Zhang, 2018; Xiao et al., 2016; Xu et al., 2021; Yu et al., 2016; Zhao et al., 2015; Zhou et al., 2016), statistical analysis approaches (Wang et al., 2023), cellular automata-Markov models (Yao et al., 2023), artificial neural networks (Wang & Zhang, 2018; Zhou et al., 2016), redundancy analysis (Zhao et al., 2015), canonical correspondence analysis (CCA), and canonical regression analysis (Luo et al., 2018, 2020; Szpakowska et al., 2022).

Several studies have shown that water nitrogen and phosphorus concentrations are positively associated with urban land use and agriculture but negatively associated with vegetation cover (Kiedrzyńska et al., 2014; Liu et al., 2012). Researchers have also found a relationship between water quality degradation, urbanization, and land use types at different scales (Wang et al., 2023; Yao et al., 2023; Li et al., 2023; Luo et al., 2020; Liu et al., 2012; Kiedrzyńska et al., 2014). Camara et al. (2019) reviewed almost 40 publications on this topic. They concluded that 87% of the studies considered urban use to be a significant source of pollution, while 82% indicated that agricultural use had a similar impact on water quality.

However, it is still unclear at what scale, whether in the entire basin or separately in parts of it, land use practices have the most significant impact on water quality (Gonzales-Inca et al., 2015). Therefore, further studies are necessary to investigate this complex relationship in more detail, especially under temporal and topographic conditions (Yu et al., 2016) and across various spatial scales (Szpakowska et al., 2022; Xu et al., 2021; Yu et al., 2016; Zhao et al., 2015). Using multitemporal data, only a few studies (Li et al., 2019; Yu et al., 2016) have examined the connections between pollutants and landscape composition in catchment areas (CAs) via field measurements. Based on our literature review, it is still unknown whether pollutants increase their concentration from the head CA to neighboring CAs in the direction of the outlet in the basin or whether the contaminant concentration is the result of a system of individual runoff and local influence for each CA according to its LULC composition.

This study investigated the relationship between the LULC composition and water quality parameters in the Los Perros River basin, located in the Isthmus of Tehuantepec in Oaxaca, Mexico. Municipal development plans indicate that the Los Perros River, which is the main water tributary, is contaminated due to waste mainly coming from homes and agriculture (Astudillo-Enriquez, 2019; Mendoza-Amézquita & Seim, 2022). Waterdrinker et al., (2020, p. 62) point out that 600 l/s of sewage enters the river. Urban settlements, contributions from agriculture, sediment drag from deforestation processes, and toxic mining waste contaminate the basin, as highlighted by Astudillo-Enríquez (2019, pp. 73–74). This situation has resulted in the loss of biodiversity, such as otters and fish, and poses risks to the health of the inhabitants (Casariego-Madorell et al., 2006). We examined whether the CAs form a sequential runoff aggregation system for certain pollutants from the basin’s highest to lowest part. Our research applied statistical (correlation, time series analysis, and canonical correspondence analysis) and geo-visual analyses to identify correlations, categorize the CAs into similar LULC groups, and identify relationships at the CA level between satellite-based LULC composition and WPI concentrations. According to the exposed, the objective of this study was to investigate how the LULC composition in the CA of each water pollution measurement station affects water pollution indicators (WPIs) of the Los Perros River basin and to investigate if the CAs form a sequential runoff aggregation system of contaminants from the highest to the lowest parts of the basin.

## Methodology

### Study area

**Fig. 1:**
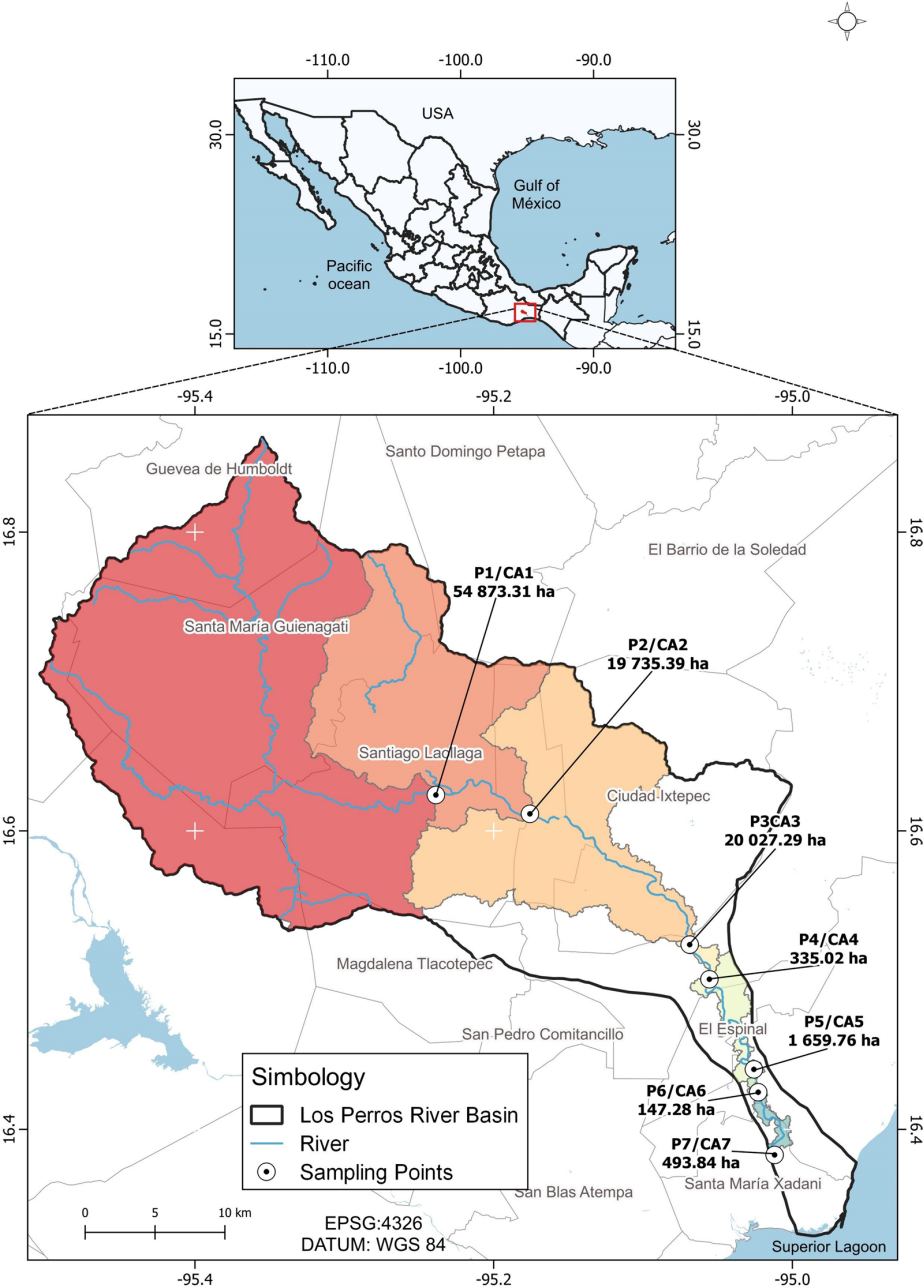
The study area, including the measurement points (P) and their CAs

The Los Perros River basin (CRLP) is a part of the “Upper and Lower Laguna Basin” in Hydrological Region number 22 (RH 22 Tehuantepec). The CRLP covers an area of approximately 1220 km^2^, and its waters flow directly into the Superior Lagoon through the territory of Santa María Xadani. The Los Perros River flows through the municipal capitals of Ixtepec, Ixtaltepec, El Espinal, Juchitán, and Santa Maria Xadani, as shown in Fig. 1. The hydrological system of the Los Perros River originates north of the urban community of Guevea de Humboldt. As it flows, it is joined on the right bank by the Santa María River in the territory of Santa María Guienagati, Arroyo Guichixú in the territory of Santiago Laollaga, Río Guigovidxi in the territory of Ixtepec City, and other small streams. On the left bank, the El Zapote River in the territory of Santa María Guienagati, the Brinca León River and the Agua Blanca Creek in the territory of Santiago Laollaga, and the Arroyo el Riito in the Santo Domingo Chihuitán territory also contribute to the river. The Los Perros River is known as the Municipality of Ixtepec city; it passes through Asunción Ixtaltepec, El Espinal, Juchitán de Zaragoza, and Santa María Xadani and ultimately flows into the Superior Lagoon.

This basin is home to many species and resources. Unfortunately, the area is also marked by high levels of marginalization and poverty (Astudillo-Enriquez, 2019; Mendoza-Amézquita & Seim, 2022). Due to the deterioration of functional dynamics and an expected increase in water pressure, this region is considered a priority area for attention (Cotler et al., 2010). The upper region of the basin is less inhabited than the lower parts and experiences more significant conservation and less human influence (Waterdrinker et al., 2020, pp. 50–51; Astudillo-Enriquez, 2019, p. 20). In 2005, towns located in the upper area of the basin did not have a drainage network, while most medium (El Espinal and Asunción Ixaltepec) and larger towns (Ixtepec and Juchitán) had this service (Astudillo-Enriquez, 2019, pp. 24–25). However, the wastewater treatment plants installed in the basin are not functional (Astudillo-Enriquez, 2019). The entry of contaminants into water bodies can be related to rain and its conduction through the surface hydrological connectivity system.

### Methodological approach

**Fig. 2:**
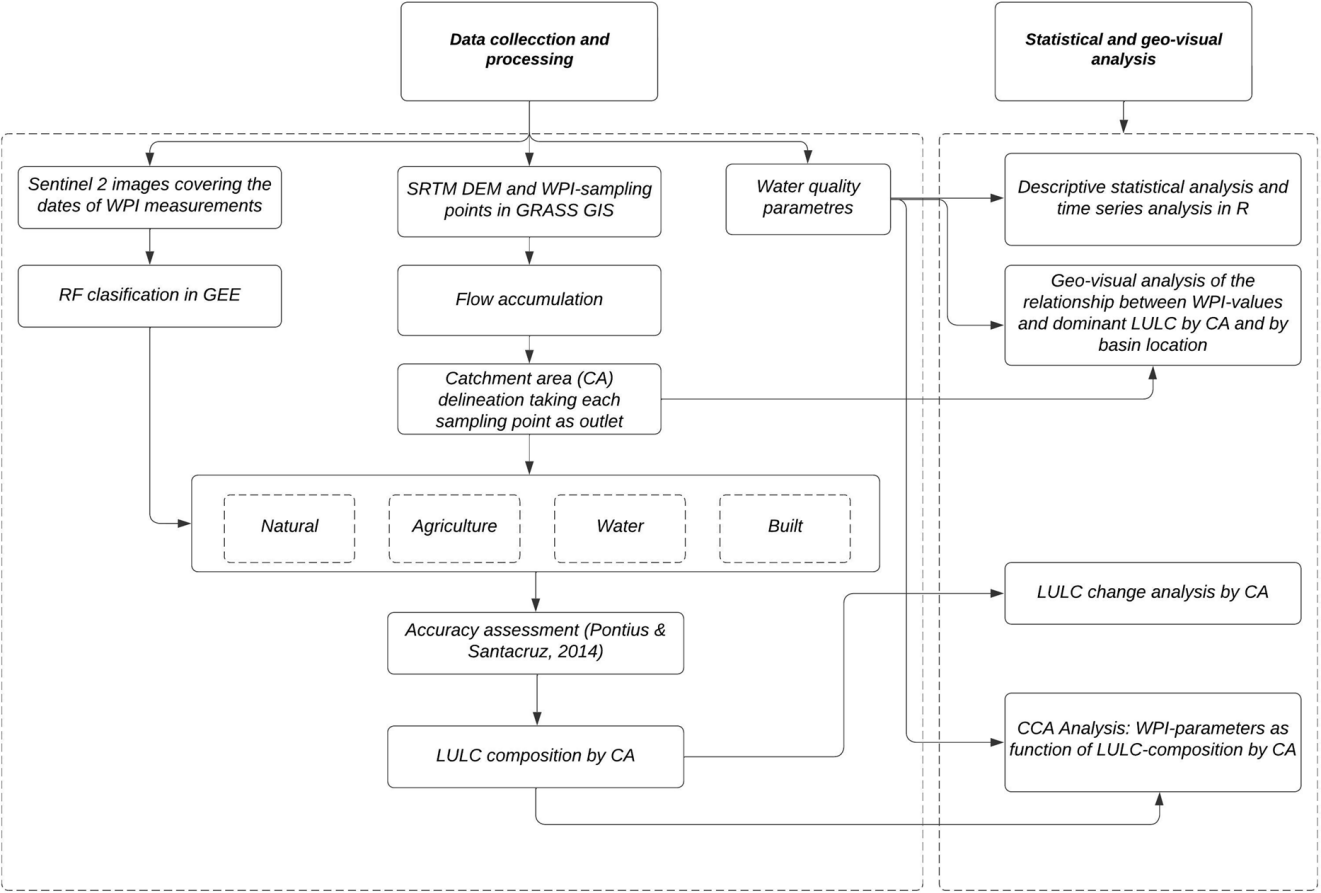
Methodological approach

Figure 2 presents the study’s methodology, which is divided into two components: data collection and processing and statistical and geo-visual analysis. The acquisition dates of the satellite images and the dates of the WPI measurements were coincident, as described in the “Data” section. As mentioned, this study’s central element was to delineate the CA of each WPI measurement point, taking it as the outlet. This approach allowed for the analysis of the effect of the LULC composition of each CA on the concentration of contaminants at each point. The contribution areas were delineated using hydrological connectivity analysis and SRTM data.

We used Sentinel 2 images and applied the random forest (RF, Breiman, 2001) classification method to determine the LULC composition, specifically the area of each LULC type by CA. For validation purposes, we calculated the sample size and assigned it to classes (Olofsson et al., 2014). Subsequently, we assessed agreement and errors (omission and commission) relative to the area (Pontius & Santacruz, 2014). Next, we used descriptive statistics, time series analysis, and geo-visual analysis to examine the WPI measurements within the basin context, considering its LULC composition. Finally, we investigated the impact of this composition on water quality using CCA. These procedures are described in detail below.

### Data

We downloaded the digital elevation model (DEM) SRTM at a resolution of 30 m from GGE using the code linked here https://code.earthengine.google.com/e50f8c15f2c3fe3e54b935e6fcb59594. Table 1 includes the measured WPIs and maximum discharge limits in national waters, defined under NOM 001-SEMARNAT-2021 (CONAGUA, 2022).

**Table 1:** The WPIs and permitted limits

WPI	Name	Abbreviation	Permitted limits (NOM 001-SEMARNAT-2021)
Physical	Conductivity	Cond	1500 µS/cm
	Potential of hydrogen	pH	6–9 UpH
	Total suspended solids	TSS	72 mg/L
	Temperature	WTemp	35 °C
Biochemical	Biochemical oxygen demand	BOD	180 mg/L
	Total phosphorus	TP	18 mg/L
	Total nitrogen	TN	30 mg/L
Bacteriological	Fecal coliforms	FC	500 NMP/100 mL

We downloaded the Sentinel 2 images and processed them on the Google Earth platform (GEE, Gorelick et al., 2017) using the code linked here https://code.earthengine.google.com/8e8a61e3f8e973697f7efa2a1dbd9e14. GEE is a cloud-based academic platform that allows for the processing of geospatial data on a global scale using Google Cloud. It hosts over 40 years of data from various satellite platforms, amounting to petabyte scales (Gorelick et al., 2017). Table 2 displays the composition dates of the selected images. The composition dates were defined to cover the sampling dates of the WPI measurements, and in the cases of 2017 and 2020, the periods of composition covered two or more field sampling dates.

**Table 2:** Dates of field measurements and composition of satellite images

Period of image composition	Date of measurements at the sampling points	Sampling points
2016–02-01 2016–03-30	30/03/2016	P1, P2, P3, P4, P5, P6
2017–03-01 2017–05-05	17/03/2017	P1, P2, P3, P4, P5, P6
	03/04/2017	P7
2018–01-01 2018–03-01	14/03/2018	P1, P2, P3, P4, P5, P6, P7
2019–03-01 2019–04-25	14/03/2019	P1, P2, P3, P4, P5, P6, P7
2020–03-01 2020–03-05	28/10/2020	P7
	31/08/2020	P3, P4, P5, P6
	30/10/2020	P1, P2

### Descriptive statistical and time series WPI analysis

We analyzed the WPI measurements to determine whether they complied with the permitted limits for rivers, streams, channels, and drains as established in NOM 001-SEMARNAT-2021 by CONAGUA (2022, Table 5 in the Appendix). The analysis considered the descriptive statistics of the WPI measurements to identify WPIs that did not comply with the norm. Descriptive statistics such as the minimum, maximum, average, standard deviation, and variance were analyzed. Then, we conducted a comparative analysis of time series at the CA level to identify the trends of the WPI measurements and to compare them with the limits using RStudio with the ggplot2 package.

### CA delineation for each measurement point

We developed the following procedure in the GrassGIS 8.2 program. The first step involved burning the DEM to ensure that the main river in the basin corresponded locally with the lowest DEM values. We implemented this process using “r.mapcalc.” Then, we obtained flow accumulations and drainage directions with a threshold value of 50,000 accumulations using the “r.watershed” command. We processed these results using the “r.water.outlet” module to delineate the CAs flowing toward each WPI measurement point.

### LULC classification and estimated area by category

We used the RF classification method to obtain four general classes related to their effect on water quality: water, natural cover, agriculture, and built areas. RF is a reliable classification method that uses predictions derived from decision trees, and it can successfully select and classify variables with the most significant capacity to discriminate between target classes (Breiman, 2001). In recent years, RF has become widely used due to the excellent classification results obtained and processing speed (Belgiu & Drăguţ, 2016). We conducted the image classification process on GGE (Gorelick et al., 2017) using the script linked here: https://code.earthengine.google.com/8e8a61e3f8e973697f7efa2a1dbd9e14.

To validate the LULC classification for every composite image, we implemented a probability sampling design chosen to achieve the priority objectives of accuracy proposed by Olofsson et al. (2014). This procedure includes calculating the sample size and assigning sampling units by strata (corresponding to each category). The method calculates the sample size using the Cochran (1977) formula for stratified random sampling. For this, values of user’s accuracies and standards error of strata need to be conjectured. After this, the sample allocation to each LULC category is performed by implementing four approaches to sample allocation: proportional, equal, optimal, and power allocation. Then, the method computes the anticipated standard errors for each sample allocation. The allocation with the lowest standard errors is selected. In our case, we calculated a sample size of approximately 1850 points were used for the studied years, and the selected sample allocation was proportional. The procedure was performed in RStudio using the Openforis Accuracy Assessment Tool (FAO, 2017). In this tool, we selected a standard error of expected overall accuracy of 0.01 and a minimum size per strata of 100 units. We exported the randomly assigned points by category to Google Earth Pro © to obtain the reference category. After this, we created the confusion matrix and calculated the accuracy indicators relative to the area, such as overall agreement, omission, and commission, using the methodology of Pontius and Santacruz (2014).

### Geo-visual analysis of WPI measurements and dominant LULC by CA

We conducted a geo-visual analysis using choropleth maps to compare the WPI average values for all the study dates with the dominant LULC by CA. This analysis was performed using QGIS 3.28.6.

### Correlation analysis of the WPIs and LULC composition by CA

We analyzed the paired correlation of the measured WPI values at each measuring point and the LULC composition by its corresponding CA using Pearson’s correlation. This analysis was implemented in R using pairs.panels from the psych package (Revelle, 2024).

### CCA

The CCA method, developed by Teer Braak (1986), is a statistical analysis method that helps define the correlation between two sets of indicators. CCA uses environmental variables (independent variables) to derive synthetic variables (ordination axes) that are related to the explanatory variables. These ordination axes are linear combinations of the independent variables and can be used to relate dependent variables to environmental variation directly. By calculating eigenvalues and eigenvectors, CCA helps us define the correlation between both sets of indicators and the degree of influence of each independent variable on each of the dependent variables (Luo et al., 2020).

This technique leads to an ordination diagram in which points represent dependent variables and sites, and vectors represent environmental variables (Ter Braak, 1986). The diagram shows the patterns of variation in the dependent variables that can be best explained by the environmental variables and approximately visualizes the “centers” of the dependent variables along each of the environmental variables (Ter Braak, 1986).

CCA and similar analytical techniques have been employed to study the impact of land use on water quality and plant composition in buffer zones adjacent to streams (Szpakowska et al., 2022). These approaches have also been used to estimate the influence of socioeconomic factors on river systems (Luo et al., 2019), assess the impact of land use and urbanization on water quality and ecology in disturbed basins (Luo et al., 2020), and analyze the effects of rapid urbanization on water quality and invertebrate communities in streams, identify the primary drivers of aquatic ecosystem dependence, and determine indicator species for fish and invertebrates (Luo et al., 2018). Furthermore, these approaches have been used to analyze the relationships among hydrological, physical, and chemical habitat environments for the potential evaluation of fish community rehabilitation in Jinan, China (Zhao et al., 2015).

We conducted CCA analyses using the R vegan package to investigate the effect of LULC composition on the measured WPI parameters at both the basin and aggregated CA levels. We assumed WPI ~ water + agriculture + natural vegetation + built-up areas. In our first CCA model, we analyzed CA1 data. We then combined these data with registers from CA2 and continued doing so until we analyzed all available data in CA7. We analyzed the data from CA4 to CA7 together because they have lower areas and different LULC compositions (higher built areas) than CA1 to CA3.

We used permutation tests to analyze whether the CCA models and their corresponding CCA terms (land cover types) and CCA axes explained more variance in the WPI value matrix than would be expected by chance. To test these hypotheses, we used the ordistep function of the vegan package of R (Blanchet et al., 2008) to perform the automatic stepwise model building of constrained ordination methods. *P* values lower than or equal to 0.05 were considered to indicate statistical significance. Finally, we interpreted the CCA ordination biplots whose models and axes were significant according to Ter Braak (1986).

## Results

This section presents the results of various analyses, including delineating CAs, classifying LULC, analyzing LULC changes within CAs, conducting statistical analysis of WPI measurements, performing geo-visual analysis of WPI values for dominant LULC by CA, and analyzing the correlation between LULC composition by CA and measured WPI values using CCA.

### WPI-descriptive statistical and time series analysis

The WPI values for each measurement point during the 5-year study are available in Table 5. Table 6 includes the table with the descriptive statistics of the measured WPIs. According to NOM 001-SEMARNAT 2021, which establishes permissible limits for contaminants in wastewater discharges in receiving bodies owned by the Mexican state (provided in Table 1), the TSS and FC values exceeded the allowed limits. The WTemp was within the permitted limit (35 °C), but in the lower part of the basin, in P6 and P7, the values closest to this limit (33.1 °C) were measured in 2019. pH values (8.7 to 9.3 UpH) around the limit (9 UpH) were measured in 2017 in the upper and middle parts of the basin (P1, P2, P3, and P4). The highest concentrations of TN (36.24 UpH, surpassing the limit of 30 mg/L), TP (5.94 mg/L), and BOD (120.94 mg/L) were measured in 2017 in the lowest part of the basin (P7). The TP and BOD measurements did not surpass the corresponding limits (18 mg/L and 180 mg/L, respectively).

**Fig. 3:**
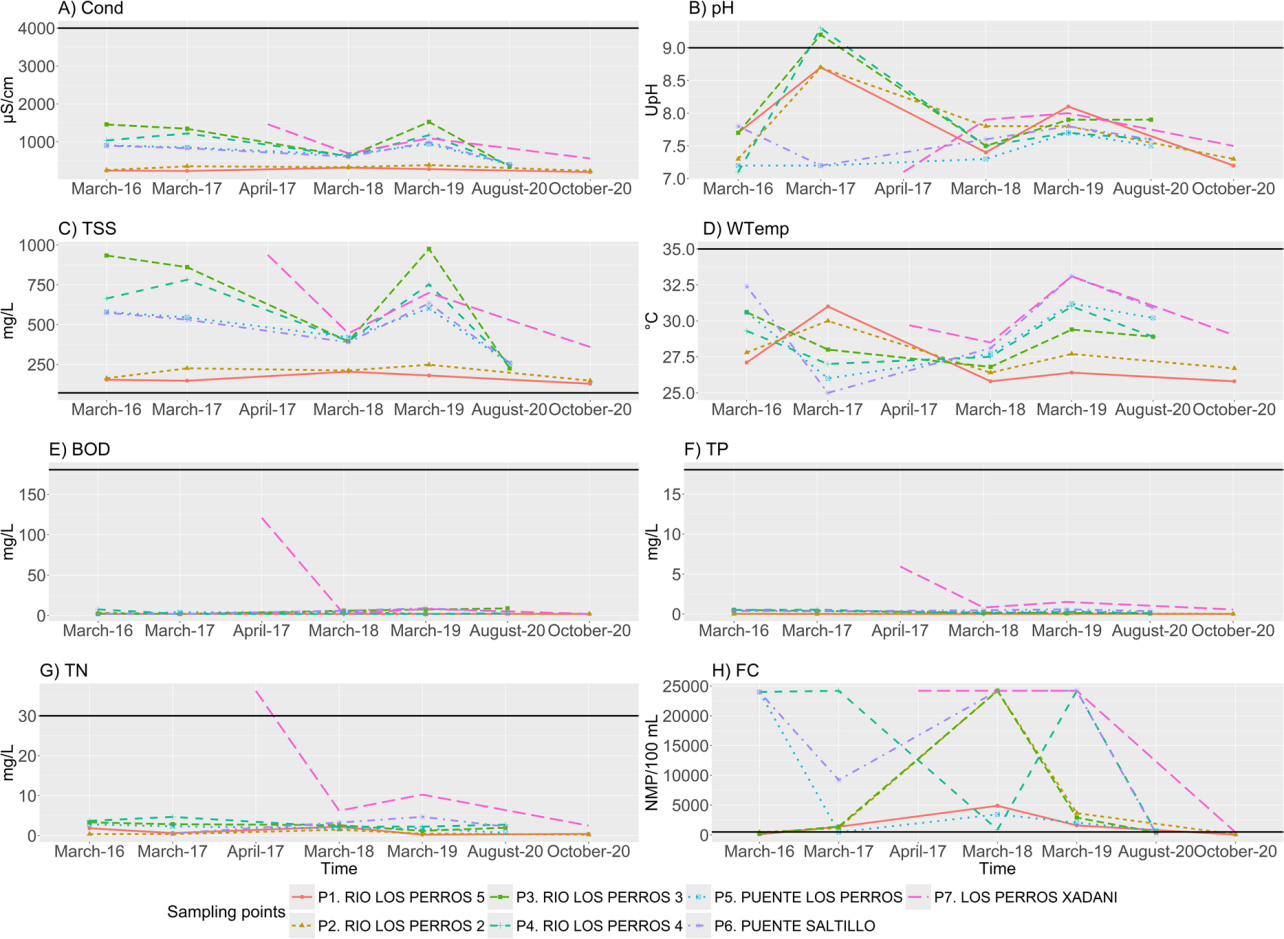
WPI-time series and corresponding limit values by measuring point (P)

The time series of the measured WPIs corresponding to the study period are shown in Fig. 3. The lowest Cond values (up to 388 µS/cm) for the entire study period were measured in the upper part of the basin (P1 and P2). The highest values (> 1500 µS/cm) were observed in March 2019 in P3, while at the other measurement points, the values fluctuated around > 1500 µS/cm for all years. However, the values of this parameter were below the permitted limit throughout the period.

In March 2017, the pH values were above the norm in the middle part of the basin (P3 and P4, 9.2 and 9.3 UpH, respectively), while values of 8.7 were present in the upper part of the basin (P1 and P2).

The TSS exceeded the established limit throughout the study period and for all stations. The values recorded in the upper part of the basin (P1 and P2) were closest to this limit during all measured days. The highest values (close to 100 mg/L) were observed for March 2016 and March 2019 in P3 and for April 2017 in P7, corresponding to the lowest part of the basin. WTemp was within the allowed limit (35 °C) for all points and dates. However, the highest temperature (33.1 °C) of this WPI was observed in March 2019 in the lower part of the basin (P6 and P7). The WTemp values increased from the highest to the lowest part of the basin, as analyzed via geo-visual analysis in the following section. In March 2017, a WTemp lower than 25 °C occurred in the lower part of the basin (P6). The other values of the other seasons and dates fluctuated around 27.5 °C.

The levels of BOD and TP found in the basin were within the allowed limits for all measurements. The highest BOD value of 120.94 mg/L was observed in April 2017 at point P7 in the lower part of the basin. The rest of the basin had values close to 0 mg/L. However, the concentration of TN found in April 2017 at point P7 in the lower part of the basin exceeded the limit, with the highest value of 36.24 mg/L. The TN concentrations recorded from the other points and dates were close to 0 mg/L.

The FC values exceeded the allowed limit for practically all dates and parts of the basin, with the highest values observed in the lower part of the basin at point P7 and in the middle part of the basin at point P3 in March 2018. The lowest values of this parameter were observed in the upper part of the basin at points P1 and P2.

### CA delineation

Figure 1 displays the CA for each measurement point, including the area for each CA. We identified seven CAs. The CAs in the upper part of the basin have larger areas than those in the middle and lower basin parts.

### Validation of the LULC classification

**Fig. 4:**
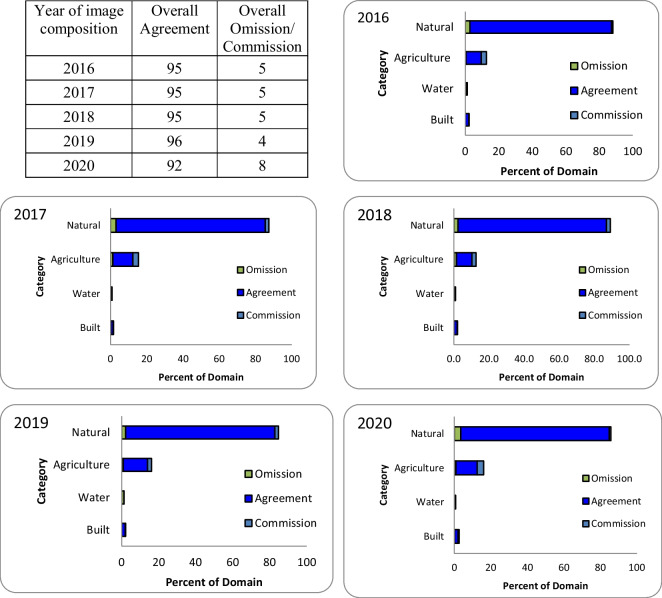
Overall omission, agreement, and commission relative to the area by category as a percentage of the domain according to Pontius and Santacruz (2014)

The results of the LULC classification validation are presented in Fig. 4. The overall agreement, relative to the area (Pontius & Santacruz, 2014), for all classified images was 92% or greater for all the studied years. As shown in the figure, natural land cover had the highest overall agreement and percentage of the domain for the studied years, followed by agriculture, built areas, and water. Water had the lowest overall agreement and percentage of the domain, with the highest omission and commission values for all the studied years.

### LULC change analysis

**Fig. 5:**
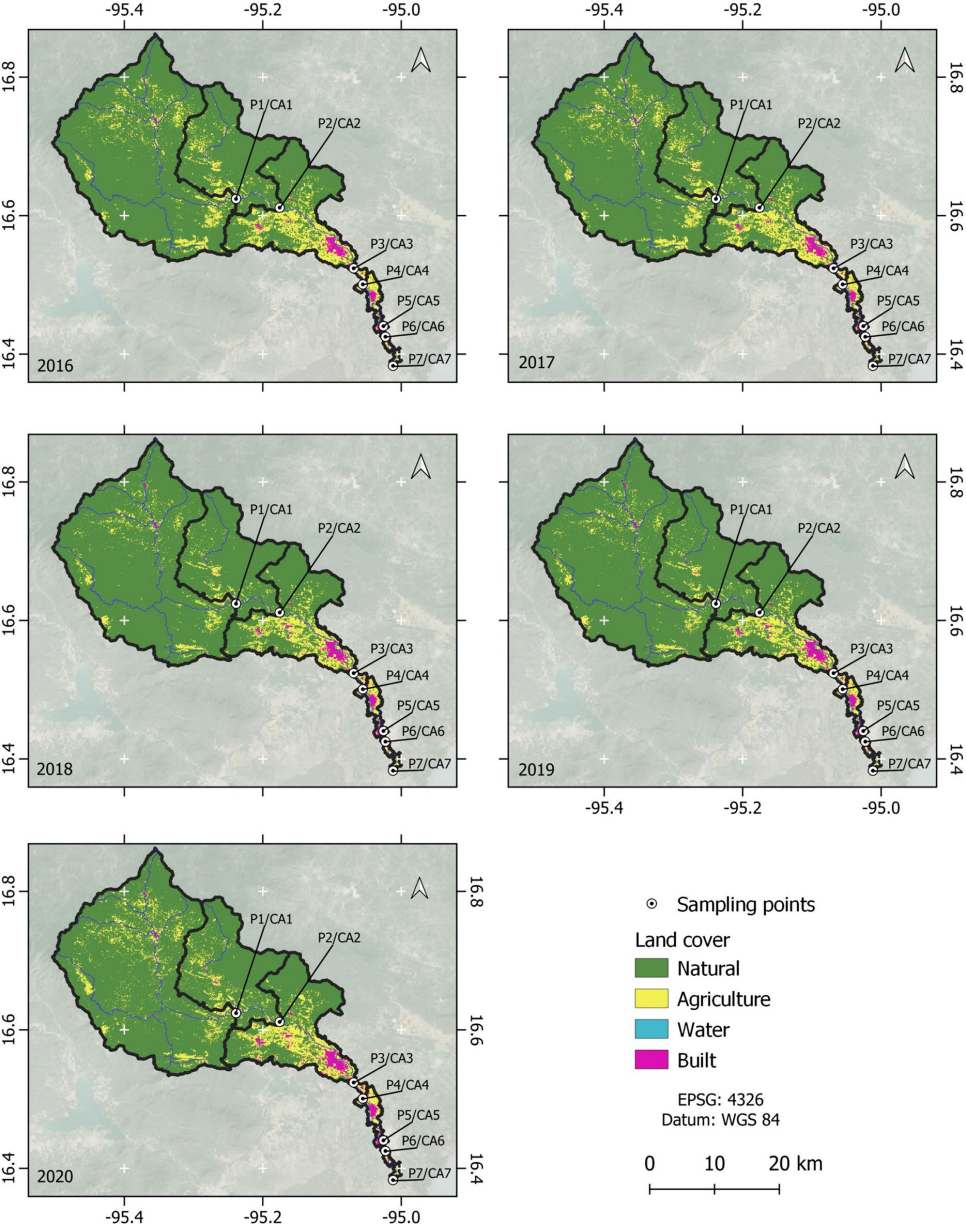
Results of the LULC classification by CA of the WPI measuring points

Figure 5 and Table 3 show the LULC classification results for each measurement point and corresponding CA over 5 years and for the whole basin. The basin covers an area of 45,349.2 ha. On average, for the 5 years studied, 84.3% were natural cover, 13.7% were agricultural land, 1.9% were built-up areas, and 0.1% were water. As shown in the table, during the study period, the natural cover decreased by 2.3% (888 ha), while agricultural land increased by 7% (439 ha), water cover increased by 12% (14 ha), and built-up areas showed the greatest increase of 67% (435 ha). This means that agricultural and built-up areas expanded into natural areas. Although the natural cover is predominant in the basin and the decrease in this type was low, it still represents an annual loss of 0.46% compared to the initial year (2016). The observed increase in water areas from 6.6 to 20.5 ha showed significant fluctuations in the intermediate years, with values higher than 80 ha, indicating strong seasonal influences on this type of LULC.

**Table 3:** The LULC variation from 2016 to 2020 for the whole basin and the CA (in ha and as % of the whole basin)

		2016		2017		2018		2019		2020	
LULC types	Area	ha	%	ha	%	ha	%	ha	2019	ha	%
Natural	CA1	22,992.6	61.4	23,806.1	61.5	24,027.2	61.1	22,992.6	61.4	22,928.9	61.4
	CA2	8231.0	22.0	8469.6	21.9	8557.0	21.7	8230.9	22.0	8170.9	21.9
	CA3	6103.7	16.3	6343.2	16.4	6600.7	16.8	6103.7	16.3	6179.8	16.5
	CA4	15.6	0.0	15.1	0.0	30.3	0.1	15.6	0.0	19.9	0.1
	CA5	77.2	0.2	67.8	0.2	113.5	0.3	77.2	0.2	50.8	0.1
	CA6	0.1	0.0	4.1	0.0	1.3	0.0	0.1	0.0	0.1	0.0
	CA7	26.7	0.1	15.7	0.0	22.3	0.1	26.7	0.1	12.2	0.0
	Whole basin	37,446.9	100.0	38,721.5	100.0	39,352.2	100.0	3,744,693.0	100.0	37,361.9	100.0
Agriculture	CA1	2542.2	37.0	1698.4	29.2	1519.4	30.1	2542.2	37.0	2606.4	37.9
	CA2	953.8	13.9	711.7	12.2	629.6	12.5	953.8	13.9	1005.2	14.6
	CA3	2693.5	39.2	2587.4	44.5	2218.4	43.9	2693.5	39.2	2545.1	37.0
	CA4	99.3	1.4	115.9	2.0	92.3	1.8	99.3	1.4	96.1	1.4
	CA5	428.7	6.2	514.7	8.8	426.5	8.4	428.7	6.2	458.3	6.7
	CA6	1.8	0.0	6.9	0.1	3.3	0.1	1.8	0.0	4.0	0.1
	CA7	144.5	2.1	182.4	3.1	161.8	3.2	144.5	2.1	167.6	2.4
	Whole basin	6863.8	100.0	5817.4	100.0	5051.3	100.0	6863.8	100.0	6882.8	100.0
Built	CA1	14.2	2.2	18.4	2.5	26.4	3.0	29.7	3.1	43.1	4.0
	CA2	7.0	1.1	7.6	1.0	11.7	1.3	16.9	1.8	25.8	2.4
	CA3	351.9	54.3	398.9	54.9	479.5	55.3	529.3	54.8	605.9	55.9
	CA4	17.7	2.7	23.4	3.2	28.8	3.3	36.2	3.7	37.8	3.5
	CA5	170.8	26.3	189.2	26.0	220.6	25.5	244.3	25.3	259.4	23.9
	CA6	56.0	8.6	57.3	7.9	60.3	7.0	62.9	6.5	63.1	5.8
	CA7	31.1	4.8	32.1	4.4	39.3	4.5	46.2	4.8	48.9	4.5
	Whole basin	648.6	100.0	726.9	100.0	866.7	100.0	965.5	100.0	1084.0	100.0
Water	CA1	2.2	33.3	60.6	72.7	10.5	13.3	18.9	25.9	5.1	24.9
	CA2	0.3	4.5	14.0	16.8	4.6	5.8	1.4	1.9	1.0	4.9
	CA3	2.2	33.3	5.6	6.7	36.5	46.2	8.6	11.8	5.1	24.9
	CA4	1.0	15.2	1.5	1.8	4.5	5.7	4.8	6.6	2.1	10.2
	CA5	0.8	12.1	1.4	1.7	12.5	15.8	22.8	31.3	4.5	22.0
	CA6	0.1	1.5	0.1	0.1	3.4	4.3	3.5	4.8	1.1	5.4
	CA7	0.1	1.5	0.1	0.1	6.9	8.7	12.9	17.7	1.6	7.8
	Whole basin	6.6	100.0	83.3	100.0	79.0	100.0	72.9	100.0	20.5	100.0

The following are the resulting percentage changes in natural coverage for each CA from 2016 to 2020: CA1 decreased by 2.3%, CA2 decreased by 1.0%, CA3 decreased by 2.6%, CA4 decreased by 17.2%, CA5 decreased by 59.5%, CA6 decreased by 98.1%, and CA7 decreased by 54.7%. These values indicate decreases of 536.9 ha, 84.6 ha, 167 ha, 6.1 ha, 74.8 ha, 6.1 ha, and 14.8 ha, respectively. The CAs with the highest percentage of natural coverage, such as CA1, CA2, and CA3, had larger areas and the lowest reduction percentages of this cover type. Conversely, the CAs with the greatest presence of built-up areas, including CA4, CA5, CA6, and CA7, had smaller area decreases but also the greatest proportions of natural coverage reduction.

Agricultural coverage underwent some changes from 2016 to 2020. CA1 increased by 24%, CA2 increased by 6.9%, CA3 decreased by 3.4%, CA4 decreased by 15%, CA5 decreased by 3.7%, CA6 decreased by 34.5%, and CA7 decreased by 2.6% (+ 505 ha, + 65 ha, 90 ha, − 17 ha, − 17.6 ha, − 2.1 ha, and − 4.5 ha, respectively). The areas with the greatest amount of natural coverage (CA1 and CA2) experienced an increase in agricultural coverage, while the others experienced a decrease.

During the entire period, the following changes were observed in the built areas: CA1 increased by 204.6%, CA2 increased by 270.1%, CA3 increased by 72.2%, CA4 increased by 113.4%, CA5 increased by 51.9%, CA6 increased by 12.8%, and CA7 increased by 57.1%. This means that the surface areas of CA1, CA2, CA3, CA4, CA5, CA6, and CA7 increased by 28.9 ha, 18.8 ha, 254.0 ha, 20.09 ha, 88.6 ha, 7.1 ha, and 17.8 ha, respectively. CA1 and CA2 showed the greatest percentage increases compared to their initial state. However, CA3 had the greatest area increase, followed by CA5.

The increases in the water cover during the study period were as follows: CA1, 135.2%; CA2, 292.3%; CA3, 129.9%; CA4, 103.9%; CA5, 502.7%; CA6, 1471.4%; and CA7, 1677.8%. The increases in ha for CA1, CA2, CA3, CA4, CA5, CA6, and CA7 were 2.9 ha, 0.8 ha, 2.9 ha, 1.1 ha, 3.8 ha, 1.0 ha, and 1.5 ha, respectively. The greatest percentage increase was observed in CA1 and CA2, followed by CA5, which had the greatest natural coverage. All CAs increased in the range of 0.8 to 3.8 ha.

### Geo-visual analysis of WPI measurements and dominant LULC by CA

In general, during the study period, the predominant land cover in the basin was natural. However, this cover type consistently decreased by almost 1% annually. At the same time, built-up and agricultural land cover steadily increased at annual rates of 10% and 33%, respectively. These trends indicate a growing human impact on the basin, which has led to the modifications mentioned above in terms of land use and land cover (LULC).

**Fig. 6:**
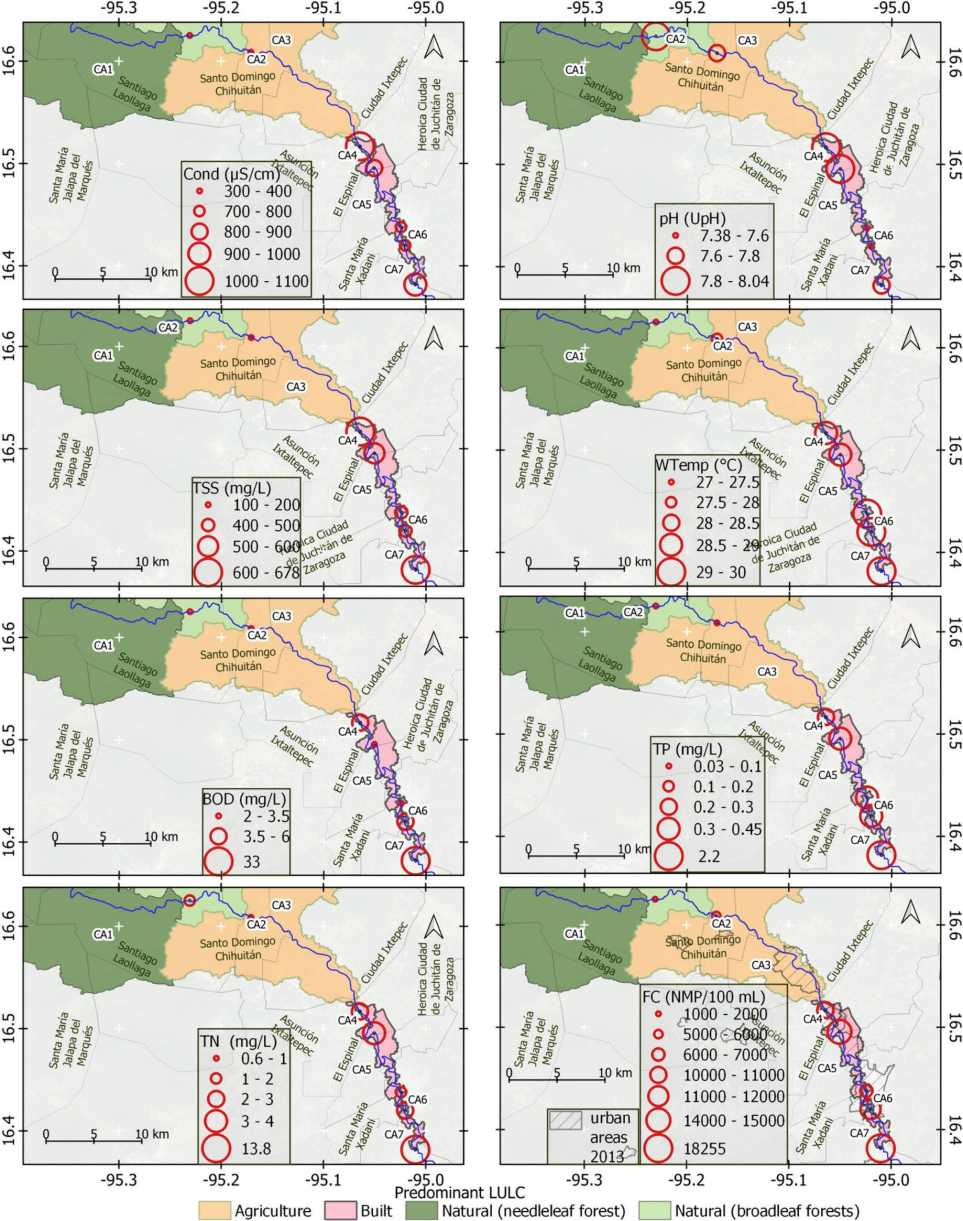
Geo-visual analysis of average WPIs (from all field measurements) related to the geographic context of the basin and concerning the dominant LULC type by CA

Figure 6 includes maps showing the average WPI values during the study period in the context of the predominant LULC type according to CA, proximity to urban areas, and location in the basin.

According to the figure, the highest levels of Cond were observed at the exits of built areas. These levels did not show accumulation patterns at the exit of the basin. The highest pH values were observed at the outlets of the CA, which were predominantly covered with vegetation, whether natural or agricultural. The highest TSS values were observed at the exit of the CAs, which had the majority of agricultural cover. Additionally, the concentration of TSS also increased at the exit of predominantly urban CAs. WTemp showed a pattern of increasing values downstream, with the lowest values at the outlet of the highest and least anthropized CA and the highest at the exit of the most urbanized CA close to the coastal zone. This may be related to the altitudinal gradient. WPIs such as BOD, TP, TN, and FC had the highest values at the outlet of the entire basin, which indicates that they followed the logic of accumulation through the drainage network toward the lowest part of the basin. Similarly, FC appeared to be associated with urban areas. Overall, the urban areas presented the highest average WPIs.

### Correlation analysis of the WPIs and LULC composition by CA

**Fig. 7:**
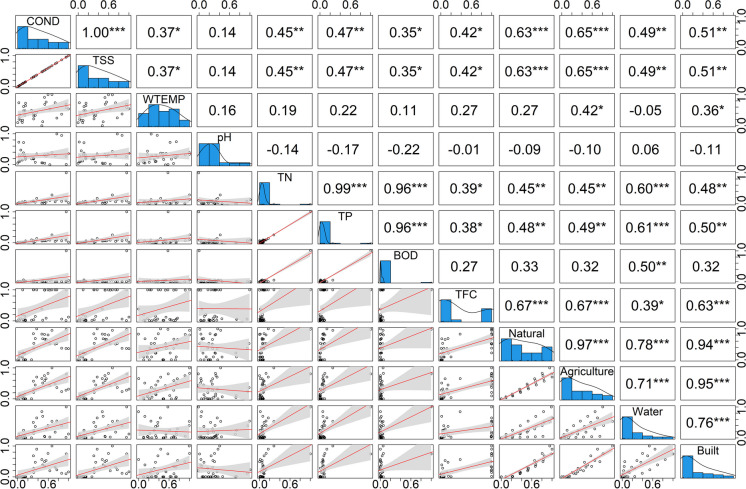
Bivariate Pearson’s correlation analysis of the associations between the measured WPI values at each measuring point and the LULC type according to CA

The results of the bivariate Pearson’s correlation analysis, used to define associations between the measured WPI values at each measuring point and the LULC composition by the corresponding CA, are presented in Fig. 7.

Cond and TSS were strongly correlated (*r* = 1). The same was observed between TP and TN (*r* = 0.99), TN and DBO (*r* = 0.96), and TP and DBO (*r* = 0.97). Water and agriculture were correlated (0.89). Cond and natural cover were correlated (*r* = 0.83), as were TSS and vegetation (*r* = 0.83). Agriculture and built covers were correlated (*r* = 0.79).

### CCA results

The results of the permutation tests of the significance of the applied models, the LC terms, and the CCA axes are shown in Table 4. The statistical significance of the CCA model for CA1 was weak. The model that included the first two CAs was significant (*p* = 0.0022); however, natural cover and vegetation, as well as the second canonical axis, were not significant. Therefore, the CCA model was considered not significant. However, the CCA models of CAs 1 to 3 were highly significant (*p* = 0.001), and the axes were significant, except for built-up areas (*p* = 0.107). Therefore, we analyzed the results of the corresponding biplot in the following section. The CCA models that included the CA 1–4 and CA 1–5 data were significant (*p* = 0.002 and *p* = 0.001, respectively). However, in both cases, only the first axis of the CCA was significant, and the terms agriculture and built areas were not significant, so these models cannot be considered statistically valid.

**Table 4:** Significance (*p*) of permutation tests for CCA under reduced models

CA	Model	LC terms	CCA-axes
P1/CA1	0.3218	0.3218	0.3218
P1/CA1 + P2/CA2	0.022**	Natural: 0.066 Agriculture: 0.127 Water: 0.019 * Built: 0.044 *	CCA1: 0.007 ** CCA2: 0.670
P1/CA1 + P2/CA2 + P3/CA3	0.001 ***	Natural: 0.001 *** Agriculture: 0.002 ** Water: 0.006 ** Built: 0.107	CCA1: 0.002** CCA2: 0.002 **
P1/CA1 + P2/CA2 + P3/CA3 + P4/CA4	0.002 **	Natural: 0.001 *** Agriculture: 0.331 Water: 0.053 Built: 0.638	CCA1: 0.004 ** CCA2: 0.360
P1/CA1 + P2/CA2 + P3/CA3 + P4/CA4 + P5/CA5	0.001 ***	Natural: 0.001 *** Agriculture: 0.228 Water: 0.011 * Built: 0.352	CCA1: 0.002 ** CCA2: 0.449
P1/CA1 + P2/CA2 + P3/CA3 + P4/CA4 + P5/CA5 + P6/CA6	0.001 ***	Natural: 0.001 *** Agriculture: 0.099 Water: 0.048 * Built: 0.050 *	CCA1: 0.001 *** CCA2: 0. 043 *
P1/CA1 + P2/CA2 + P3/CA3 + P4/CA4 + P5/CA5 + P6/CA6 + P7/CA7	0.001 ***	Natural: 0.001 *** Agriculture: 0.156 Water: 0.002 ** Built: 0.041 *	CCA1: 0.001 *** CCA2: 0.003 **
P4/CA4 + P5/CA5 + P6/CA6 + P7/CA7	0.001 ***	Natural: 0.001 *** Agriculture: 0.289 Water: 0.001 *** Built: 0.110	CCA1: 0.001 *** CCA2: 0.101

Key: < 0.001: ***; 0.001: **; 0.01: *; 0.05: “.”; > 0.1: “”

The CCA including CA 1 to 6 data showed statistical significance (*p* = 0.001) along with its axes and terms, except for agriculture (*p* = 0.099). This model, which was considered statistically valid, is further analyzed in the following section. Another CCA model, quite similar to the previous one, was the CCA of CAs 1 to 7 (which corresponds to the entire basin). This model, which was also statistically significant (*p* = 0.001), is discussed in the following section. The term agriculture (*p* = 0.156) was the only exception. The last CCA model for CAs 4 to 7 was statistically significant (*p* = 0.001), but only two of its four terms were significant, and its axes were not significant. Therefore, this model is considered to be statistically nonsignificant.

#### Significant CCA results

**Fig. 8:**
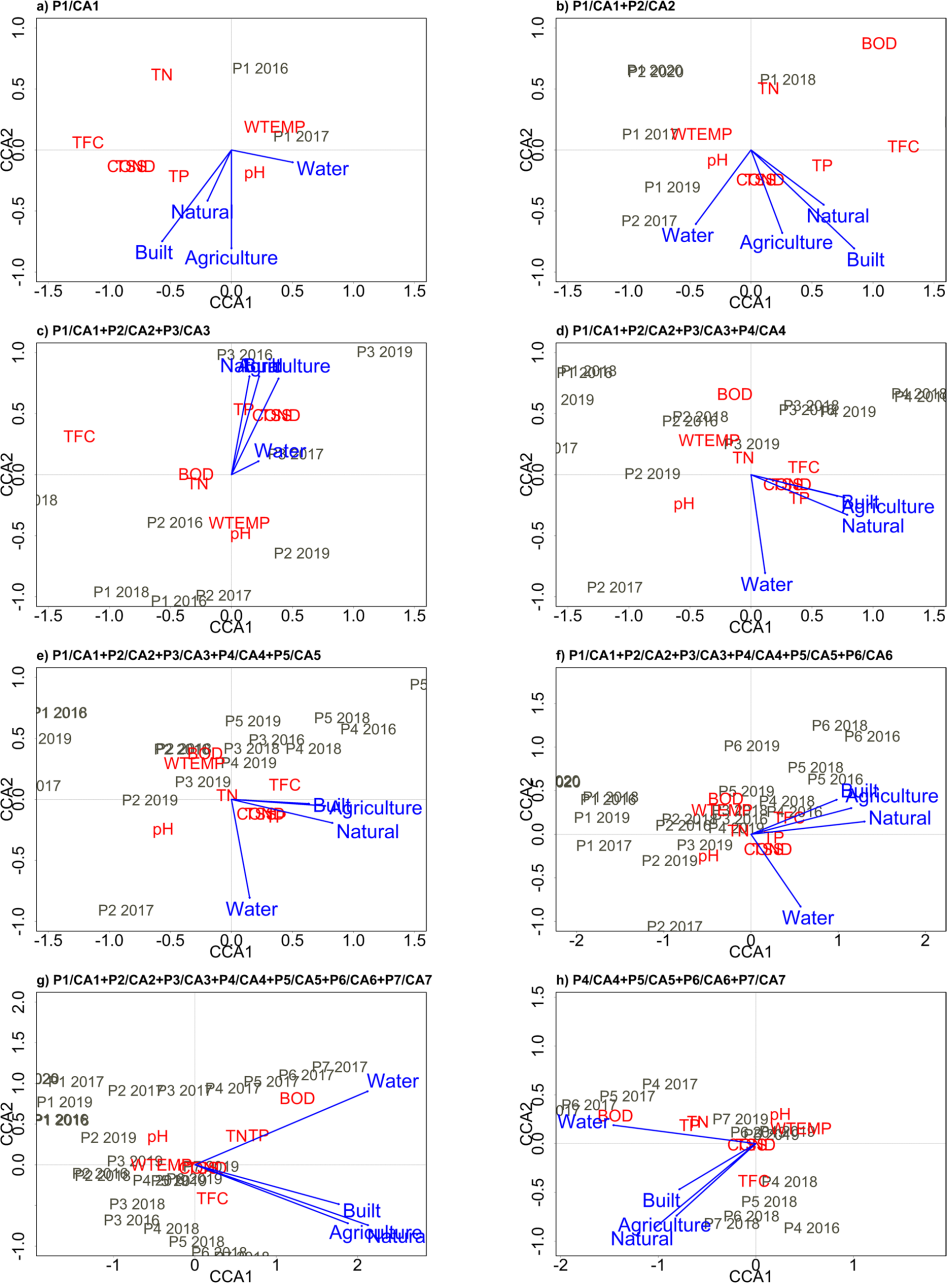
CCA ordination plots by CA group. The following formula was applied: WPI ~ natural + agriculture + water + built-up

The CCA ordination plots of the eight CA groups, as indicated in the methodology section, are shown in Fig. 8. According to Ter Braak (1986), in the diagram, the sites and WPI measurements are represented by their location in the ordination diagrams, the LULC composition by CA is represented by arrows, and the WPI measurement locations and the arrows of the LULC composition jointly reflect the WPI measurement distributions along each of the LULC composition variables, which are considered the environmental variables. In the following paragraphs, only the significant results of the CCA models are analyzed.

##### *CCA for P1/CA1 + P2/CA2 + P3/CA3*

The resulting ordination diagram is shown in Fig. 8c. The first CCA axis explained 52.7% of the variance, while the second axis explained 41.9%, for a cumulative proportion of 94.7%. The diagram indicates that Cond and TSS were mutually correlated, as they were collocated. Moreover, the highest WPI measurements were not correlated with P1 or P2. These measurement points correspond to CA1 and CA2, which are the parts of the basin with the highest natural cover area. In contrast, the WPI measurements appeared close to the measurements at P3, which is the outlet of CA3 and had the highest area of human-related LULC cover compared to CA2 and CA1.

The highest weighted average of TP, Cond, and TSS concerning agriculture, natural, and built covers indicate that the highest values of these WPI measurements occurred in CA with those kinds of predominant cover. According to the same criteria, BOD and TN were the next most common, followed by FC, WTemp, and pH, in terms of occurrence in CA, with a predominance of these land cover types. Water appeared to be less correlated with the CCA axis than with the other cover types, but its correlation with the WPI parameters was similar to that of the other cover types.

##### *CCA for P1/CA1 + P2/CA2 + P3/CA3 + P4/CA4 + P5/CA5 + P6/CA6*

Figure 8f displays the resulting ordination diagram of this analysis. The first CCA axis explained 65.7% of the variance, while the second axis explained 27.2%, for a cumulative proportion of 93%. In the ordination diagram, Cond and TSS appeared to be mutually correlated given that they are close to each other. For these six aggregated CAs, the highest WPIs are associated with P5, P4, and P6. These measurement points corresponded to the CAs with the highest human-related LULC cover. The highest weighted average of FC, P, Cond, and TSS concerning agriculture, built, and natural covers indicated that the highest values of these WPI measurements occurred in CA with those kinds of predominant cover. BOD, TN, WTemp, and pH were next in terms of occurrence in CA, with a predominance of these land cover types using the same criteria.

Water cover behaved differently in terms of its relationship with the WPI measurements. The highest TSS, Cond, pH, and TP values (in this order) appeared to occur within CA, which had the highest water areas, whereas the lowest values related to this cover type were DBO, WTemp, TN, and FC.

##### *CCA for P1/CA1 + P2/CA2 + P3/CA3 + P4/CA4 + P5/CA5 + P6/CA6 + P7/CA7*

The resulting ordination diagram, shown in Fig. 8g, represents the entire basin (the aggregation of all the CAs). The first CCA axis explained 58.9% of the variance, and the second axis explained 29.2%, for a cumulative proportion of 88.2%. As seen in the ordination diagram, Cond and TSS appeared to be mutually correlated because they are collocated. The diagram also shows a general grouping of P1, P2, and P3 on one side and P4, P5, P6, and P7 on the other. The built, agricultural, and natural cover had similar biplot scores, while the water cover remained separate in the plot. The diagram reveals that the highest WPI values of DBO, TP, FC, Cond, and TSS were correlated with P4, P5, P6, and P7, which corresponded to the most anthropized CAs. The highest weighted averages of DBO, TP, TN, FC, Cond, and TSS (in that order) with respect to agriculture, natural, and built covers indicate that the highest values of these WPI measurements occurred in CA with those kinds of predominant cover.

## Discussion

In this research, we analyzed at what scale (whole basin or aggregated ACs) the LULC composition obtained by image classification most influenced the values of the WPIs. To do this, we analyzed the WPI measurements, obtained the LULC for each AC of the basin, analyzed these values in a spatial context, and applied bivariate correlation analysis and CCA to define the correlations between the LULC and WPI variables. We present an innovative approach in this study given that the study area is divided into ACs for aggregate analysis (according to the basin approach) of the relationship of the LULC with the contributions of contaminants coming from the types of land use, taking the WPI measurement points as the outlet of each AC. In the following paragraphs, we discuss aspects of the results obtained from these processes developed in the research.

We calculated the sample number to validate our satellite data classification results, assigned it to classes following Olofsson et al. (2014), and estimated the agreement and errors (overall and by category following Pontius & Santacruz, 2014). This approach allowed for reliable validation. The overall agreement (Pontius & Santacruz, 2014) for all classified images was 92% or greater for all the studied years. The natural cover area had the highest agreement and percentage of the domain (s. Figure 4). In contrast, water and built areas had the lowest percentages of domains and the highest omission errors. These results explain why the overall agreement was high. However, assigning the sample size in the LULC classes can introduce some subjectivity by starting with an estimate by category of the error (Ui) that the user proposes based on the possible confusion that this class may present in the classification. However, we consider our classification reliable according to the relevant literature (Olofsson et al., 2014; Pontius & Santacruz, 2014).

The predominant land cover in the basin was natural, but this cover type decreased by 1% annually. Conversely, built-up and agricultural land cover increased at 10% and 33% annual rates, respectively. These results indicate a growing human impact on the basin, which affected the WPI, as noted in the following discussion.

The TSS and FC values exceeded the allowed limits for all the ACs and dates (s. Figure 3c, h). As Lewis et al. (2013) noted, rainfall generates flow paths connecting upland microbial pollution sources, including suspended particulates. Paule-Mercado et al. (2022) reported that increases in bare land areas and impervious cover in the basin lead to increased fecal contamination and TSS. Accordingly, in this study, the observed decrease in natural cover and increase in built-up and agricultural cover in the upper CAs of the study basin (s. the “LULC change analysis” section) may be related to these elevated WPIs. Paule-Mercado et al. (2022) reported that TSS is the strongest predictor of fecal indicator bacterial dynamics. We found the lowest FC values in the upper part of the basin at points P1 and P2. Given that the CAs of these points also have the lowest built area in the basin, the concentration of FC can be linked to untreated wastewater discharge from urban areas into the river.

Our study did not find a correlation between WTemp and pH, as other authors did (e.g., Yang et al., 2018). In our study, these values usually complied with the permitted limits. The pH values above the norm measured in March 2017 in the middle of the basin may correspond to an exceptional event because the values decreased to 7–8 UpH immediately after this date.

Some measured values indicate adverse effects on aquatic life in the lower part of the basin (CA7/P7). In April 2017, TN levels exceeded the limit, while TP and BOD had the highest measured values. The concentrations of these WPIs in the lowest part of the basin suggest that they accumulated from the upper CA through runoff. The geo-visual analysis of average WPI values vs. dominant LULC also revealed this accumulation: WPIs such as BOD, TP, TN, FC, and Wtemp exhibited the highest values at the outlet of the entire basin, indicating accumulation through the drainage network toward the lowest part of the basin. Generally, all measured WPIs decreased from March 2019 to October 2020, possibly due to the COVID-19 pandemic and its impact on the reduction in anthropogenic activity.

A lack of WQA measurement records and corresponding LULC for a specific date may have caused the CCA model of CA1 to fail to achieve statistical significance. However, a statistically significant model was obtained for the aggregate analysis of CAs 1 to 3, whose axes and terms were also statistically significant except for built areas (because they represent a reduced percentage of the total area of these CAs). This result suggested that the WPIs that responded to the composition of LULC in these CAs were mainly dominated by natural vegetation and agriculture. This part of the basin is less inhabited and has more significant conservation and less human influence than CAs 4 to 7, which have much less coverage of these LULC types. In addition, the CCAs of CAs 1 to 4 and 1 to 5 were statistically invalid. However, the CCAs that included P/CAs 1 to 6 and those that included P/CAs 1 to 7 (representing the entire basin) were significant. These results indicated that it is possible to establish a function in terms of runoff and accumulation of contaminants according to the logic of surface hydrological connectivity at the basin level. It also shows that the dataset used in the research allows for defining relationships between LULC and WPIs.

CCA analysis of the last group of CAs (4 to 7) did not reveal a significant correlation. These CAs primarily included urban areas, and factors other than LULC could impact water quality. For instance, rivers may receive contaminants from various sources, such as households, businesses, and industries that discharge untreated waste. Zhou et al. (2016) reported that point source pollution weakens the land use-water quality correlation. In the studied basin, some urban communities cannot access drainage services and dispose of their waste in the open air or the river. Additionally, wastewater treatment plants installed in the basin are not functional (Astudillo-Enriquez, 2019).

Based on the results of the significant CCA models, the first two axes of the CCA model of the records of CAs 1 to 3 explain almost all the variability (97.9%). Therefore, these axes are adequate for establishing the connection between the values of the WPIs and the composition in terms of the LULC of each CA. The ordination diagram shows that the environmental variables agriculture and built areas were located close to each other and separated from the water areas. This observation reveals the different impacts of areas with more human activity and water bodies on water quality. High levels of BOD, TP, TN, and FC appeared for the entire basin to be more related to built and agricultural areas. Our results concerning the relationship between agricultural areas with the highest TP and TN concentrations coincide with those analyzed at the CA level by Li et al. (2019). Other studies that agree that TP is associated with agricultural areas include those by Kiedrzyńska et al. (2014). In agreement with our study, Li et al. (2019) found a high correlation between BOD and urban CA at the CA level.

The WPIs of areas with relatively high amounts of natural cover were similar to those of agricultural and built areas for all the significant CCAs (CAs 1 to 3, 1 to 6, and 1 to 7). This unexpected result may be related to the TSS and FC exceeding the limits in all the CAs and dates. The observed decrease in natural cover and increase in built-up and agricultural cover in the upper CAs of the study basin possibly contributed to this result. However, the measurement points appeared differentiated in the ordination diagram (Fig. 6), grouping those corresponding to CA with little anthropogenic activity and those with preponderant human influence. Therefore, we considered the relationships of CAs with significant human activity and greater pollutant concentrations to be valid. However, further investigation is needed to determine why CAs with a natural preponderance have similar scores in the biplot to those of agricultural and built coverage.

In general, all the results of the CCA obtained in our study coincide with those of Wang et al. (2023), Luo et al. (2020), and Luo et al. (2018), who found that water quality is negatively affected by land use, particularly agriculture and urbanization. Another study carried out at the subbasin level (Yu et al., 2016) concluded that all high WPI concentrations were significantly correlated with agricultural, forestry, grassland, and urban areas at all the stations analyzed. The statistical significance and high correlation of the axes with the terms (LULC variables) of the CCA model found in our study for the aggregation of all the CAs of the study basin (corresponding to the CCA of CA1 + CA2 + CA3 + CA4 + CA5 + CA6 + CA7) allows us to affirm that these variables considerably determine the WPI values at the basin level.

## Conclusions

The observed decrease in natural cover and increase in built-up and agricultural cover in the upper CAs of the study basin between the study period (2016 to 2020) could be related to elevated WPI values of TSS and FC values, which exceeded the allowed limits on all CA and measured dates. Another piece of evidence that anthropogenic activity strongly influences the WPI is that all measured values decreased from March 2019 to October 2020 (the COVID-19 pandemic).

We discovered a significant correlation between the LULC composition and the concentration of contaminants in the entire basin. This connection is strong in regions where agriculture and natural vegetation cover a large portion of the land. We also observed that areas with intense human activity, such as urban and agricultural zones, have different impacts on water quality than those with more natural coverage, such as bodies of water and natural vegetation.

Moreover, we found that when we analyzed CAs with urban predominance separately, the LULC composition was not significantly related to the WPI-measured values. This indicates that there may be other factors, such as wastewater discharge from residential and industrial areas or waste dumps into the riverbed, which can determine the variability of these values.

We observed that it is important to analyze basins by dividing them into CAs. This is because some CAs may undergo different processes in terms of LULC composition and other factors that can affect the concentration of contaminants in the water. WPIs such as BOD, TP, TN, FC, and Wtemp exhibited the highest values at the outlet of the entire basin, indicating accumulation through the drainage network toward the lowest part of the basin. Accordingly, CAs form a sequential aggregation system of these pollutants from the highest to the lowest part of the basin.

Our study also confirmed that satellite data sources to define LULC composition by CA and specific WPI measurements on the outlets of these CAs are useful tools for studying, monitoring, and remedying the effects of human activity on the water quality of basin rivers. This approach can help prevent the loss of various species, such as otters and different fish, and prevent health risks to inhabitants.

## Data Availability

Data is provided within the manuscript by mean of Google Earth Engine links.

## Appendix

**Table 5:** The permitted limits for rivers, streams, channels, and drains as established in NOM 001-SEMARNAT-2021 by CONAGUA (2022)

Name of measuring point	Date	COND	TSS	WTemp	pH	TN	TP	BOD	TFC
P1. RIO LOS PERROS 5	30/03/2016	241	**154.24**	27.1	7.7	1.84858	0.031738	2	93
P1. RIO LOS PERROS 5	17/03/2017	231	**147.84**	31	8.7	0.63875	0.02001	2	**1379**
P1. RIO LOS PERROS 5	14/03/2018	320	**204.8**	25.8	7.4	2.22304	0.02827	2	**4884**
P1. RIO LOS PERROS 5	14/03/2019	282	**180.48**	26.4	8.1	0.22552	0.03185	2	**1597**
P1. RIO LOS PERROS 5	30/10/2020	201.8	**129.15**	25.8	7.2	0.423	0.019	2	30
P2. RIO LOS PERROS 2	30/03/2016	255	**163.2**	27.8	7.3	0.39046	0.026519	3.4	430
P2. RIO LOS PERROS 2	17/03/2017	353	**225.92**	30	8.7	0.39291	0.02103	2	**1153**
P2. RIO LOS PERROS 2	14/03/2018	331	**211.84**	26.4	7.8	1.47653	0.07042	4.68	**24196**
P2. RIO LOS PERROS 2	14/03/2019	388	**248.32**	27.7	7.8	0.48923	0.05228	2	**3654**
P2. RIO LOS PERROS 2	30/10/2020	231.6	**148.22**	26.7	7.3	< 0.231	0.025	< 2	90
P3. RIO LOS PERROS 3	30/03/2016	1459	**933.76**	30.6	7.7	3.31584	0.51646	2.3	210
P3. RIO LOS PERROS 3	17/03/2017	1345	**860.8**	28	**9.2**	2.85891	0.40267	2	**1401**
P3. RIO LOS PERROS 3	14/03/2018	617	**394.88**	26.8	7.5	2.62597	0.17919	5.94	**24196**
P3. RIO LOS PERROS 3	14/03/2019	1524	**975.36**	29.4	7.9	1.21103	0.13678	7.48	**2909**
P3. RIO LOS PERROS 3	31/08/2020	352	**225.28**	28.9	7.9	1.9642	0.0812	8.88	430
P4. RIO LOS PERROS 4	30/03/2016	1038	**664.32**	29.3	7.1	3.70637	0.515815	7.6	**24000**
P4. RIO LOS PERROS 4	17/03/2017	1221	**781.44**	27	**9.3**	4.68075	0.54495	2	**24196**
P4. RIO LOS PERROS 4	14/03/2018	620	**396.8**	27.5	7.5	2.23094	0.06469	2	**884**
P4. RIO LOS PERROS 4	14/03/2019	1177	**753.28**	31	7.7	2.25126	0.28289	2	**24196**
P4. RIO LOS PERROS 4	31/08/2020	391	**250.24**	28.9	7.6	2.7546	0.1165	2.85	430
P5. PUENTE LOS PERROS	30/03/2016	906	**579.84**	30.6	7.2	2.83102	0.452577	2.1	**24000**
P5. PUENTE LOS PERROS	17/03/2017	853	**545.92**	26	7.2	2.30723	0.36982	4	448
P5. PUENTE LOS PERROS	14/03/2018	658	**421.12**	27.7	7.3	1.79504	0.24825	4.1	**3448**
P5. PUENTE LOS PERROS	14/03/2019	941	**602.24**	**31.2**	7.7	1.83982	0.38081	2	**2046**
P5. PUENTE LOS PERROS	31/08/2020	402	**257.28**	30.2	7.5	0.7054	0.1825	2.61	**750**
P6. PUENTE SALTILLO	30/03/2016	899	**575.36**	32.4	7.8	0	0.394905	2	**24000**
P6. PUENTE SALTILLO	17/03/2017	830	**531.2**	25	7.2	0.57544	0.34885	2	**9208**
P6. PUENTE SALTILLO	14/03/2018	609	**389.76**	28.1	7.6	3.27619	0.47464	5.96	**24196**
P6. PUENTE SALTILLO	14/03/2019	991	**634.24**	**33.1**	7.8	4.69364	0.5908	9.01	**24196**
P7. LOS PERROS XADANI	03/04/2017	1466	**938.24**	29.7	7.1	**36.2423**	5.93706	120.94	**24196**
P7. LOS PERROS XADANI	14/03/2018	696	**445.44**	28.5	7.9	6.23312	0.82351	2	**24196**
P7. LOS PERROS XADANI	14/03/2019	1094	**700.16**	**33.1**	8	10.2507	1.51369	8.26	**24196**
P7. LOS PERROS XADANI	28/10/2020	561	**359.04**	29	7.5	2.515	0.568	2	430

The bold highlighted values are over the permitted limits

**Table 6:** Descriptive statistics of the WPIs

Measuring point	QWI	Minimum	Maximum	Average	Standard deviation	Variance
P1	COND	201.8	320.0	255.2	46.25	2139.03
	TSS	129.2	204.8	163.3	29.6	876.15
	WTemp	25.8	31.0	27.22	2.18	4.75
	Ph	7.2	8.7	7.82	0.6	0.36
	TN	0.22	2.22	1.07	0.9	0.81
	TP	0.02	0.03	0.03	0.01	0.0
	BOD	2.0	2.0	2.0	0	0.0
	TFC	30.0	4884.0	1597.0	1972.9	3892349.3
P2	COND	231.6	388.0	311.7	66.2	4382.69
	TSS	148.2	248.3	199.5	42.37	1795.15
	WTemp	26.4	30.0	27.72	1.41	2.0
	Ph	7.3	8.7	7.78	0.57	0.33
	TN	0.23	1.48	0.60	0.5	0.25
	TP	0.02	0.07	0.04	0.02	0.0
	BOD	2.0	4.68	2.82	1.21	1.45
	TFC	90.0	24196.0	5905.0	10319.87	106499758.8
P3	COND	352.0	1524.0	1059.0	536.94	288308.3
	TSS	225.3	975.4	678.0	343.64	118091.08
	WTemp	26.8	30.6	28.74	1.43	2.06
	Ph	7.50	9.2	8.04	0.67	0.45
	TN	1.21	3.32	2.40	0.82	0.68
	TP	0.08	0.52	0.26	0.19	0.03
	BOD	2.00	8.88	5.32	3.08	9.47
	TFC	210.0	24196.0	5829.0	10322.34	106550658.7
P4	COND	391.0	1221.0	889.4	365.97	133933.3
	TSS	250.2	781.4	569.2	234.22	54859.08
	WTemp	27.0	31.0	28.74	1.58	2.5
	Ph	7.1	9.3	7.84	0.85	0.72
	TN	2.23	4.68	3.13	1.06	1.11
	TP	0.06	0.54	0.3	0.22	0.05
	BOD	2.0	7.6	3.29	2.44	5.94
	TFC	430.0	24196.0	14741.0	12858.31	165336075.2
P5	COND	402.0	941.0	752.0	224.15	50243.5
	TSS	257.3	602.2	481.3	143.46	20579.74
	WTemp	26.0	31.2	29.14	2.2	4.86
	Ph	7.2	7.70	7.38	0.22	0.05
	TN	0.71	2.83	1.90	0.79	0.62
	TP	0.18	0.45	0.33	0.11	0.01
	BOD	2.0	4.10	2.96	1.02	1.04
	TFC	448.0	24000.0	6138.0	10055.32	101109562.8
P6	COND	407.0	991.0	747.2	236.73	56043.2
	TDS	260.5	634.2	478.2	151.51	22955.29
	WTemp	25.0	33.10	29.9	3.34	11.19
	Ph	7.2	7.80	7.6	0.24	0.06
	TN	0.0	4.69	2.16	1.93	3.71
	TP	0.35	0.59	0.44	0.1	0.01
	BOD	2.0	9.01	4.74	2.95	8.68
	TFC	230.0	24196.0	16366.0	11096.21	123125804.0
P7	COND	561.0	1466.0	954.2	409.37	167585.58
	TSS	359.0	938.2	610.7	261.99	68643.05
	WTemp	28.5	33.1	30.07	2.08	4.31
	Ph	7.1	8.0	7.63	0.41	0.17
	TN	2.52	36.24	13.81	15.28	233.62
	TP	0.57	5.94	2.21	2.52	6.33
	BOD	2.0	120.94	33.3	58.5	3422.38
	TFC	430.0	24196.0	18255.0	11883	141205689.0

Values rounded to two decimals
